# Genetic influence on family socioeconomic status and children's intelligence^☆^

**DOI:** 10.1016/j.intell.2013.11.002

**Published:** 2014-01

**Authors:** Maciej Trzaskowski, Nicole Harlaar, Rosalind Arden, Eva Krapohl, Kaili Rimfeld, Andrew McMillan, Philip S. Dale, Robert Plomin

**Affiliations:** a King's College London, MRC Social, Genetic and Developmental Psychiatry Centre, Institute of Psychiatry, De Crespigny Park, London, SE5 8AF, United Kingdom; b Department of Psychology and Neuroscience, University of Colorado Boulder, 345 UCB, Boulder, CO 80309, United States; c Department of Speech and Hearing Sciences, University of New Mexico, 1700 Lomas Blvd, NE Suite 1300, Albuquerque, NM 87131, United States

**Keywords:** SES, Socioeconomic status, Intelligence, Cognitive abilities, GCTA

## Abstract

Environmental measures used widely in the behavioral sciences show nearly as much genetic influence as behavioral measures, a critical finding for interpreting associations between environmental factors and children's development. This research depends on the twin method that compares monozygotic and dizygotic twins, but key aspects of children's environment such as socioeconomic status (SES) cannot be investigated in twin studies because they are the same for children growing up together in a family. Here, using a new technique applied to DNA from 3000 unrelated children, we show significant genetic influence on family SES, and on its association with children's IQ at ages 7 and 12. In addition to demonstrating the ability to investigate genetic influence on between-family environmental measures, our results emphasize the need to consider genetics in research and policy on family SES and its association with children's IQ.

## Highlights

• Twin research shows genetic influence on within-family environments.
• Twin research cannot address genetic influence on children's family-level SES.
• A new DNA-based method (GCTA) shows genetic influence on family-level SES.
• GCTA shows that genes drive the correlation between family SES and children's IQ.
• Heritability can be viewed as an index of meritocratic social mobility.

## 1 Introduction

A surprising finding from quantitative genetic research is that most environmental measures in the social and behavioral sciences show significant and substantial genetic influence (Kendler and Baker, 2007, Plomin, 1994, Plomin and Bergeman, 1991, Vinkhuyzen et al., 2010). This genetic influence on environmental measures is attributed to genotype–environment correlation in which individuals' experiences are correlated with their genetic propensities (Plomin, DeFries, Knopik, & Neiderhiser, 2013). Most of this quantitative genetic research relies on the classical twin design that compares monozygotic and dizygotic twins. However, using the twin method for this purpose runs into a major limitation, especially in developmental studies: The twin design can only investigate environmental factors that make members of a twin pair living in the same family *different* from one another, called within-family environmental effects. However, some of the most influential aspects of the family environment operate between rather than within families. For example, consider the most widely studied measure in the social and behavioral sciences, socioeconomic status (SES), which we refer to as family SES because of our focus on children's development (Bradley & Corwyn, 2002). A study of school-age twins cannot detect genetic influence on family SES or its effect on twins' cognitive development because family SES is the same for members of a twin pair. Because family SES is the same for both twins in a family, a twin study would mistakenly attribute variance in family SES to shared environment even if genetic factors were in fact substantially involved. Most importantly, genetic mediation of the effect of family SES on children's cognitive development would also be missed by the twin method.

A new quantitative genetic method that uses DNA from unrelated individuals can solve this problem because it can assess genetic effects on between-family and between-school differences in children's outcomes. The method, called *Genome-wide Complex Trait Analysis (GCTA)*, foregoes the identification of individual DNA variants to estimate the total genetic influence captured by genome-wide genotyping for a large sample of unrelated individuals whose genetic similarity is compared pair by pair (Yang, Lee, Goddard, & Visscher, 2011). The significance of GCTA is that it can estimate the net effect of genetic influence using DNA of unrelated individuals rather than using familial resemblance in groups of special family members who differ in genetic relatedness such as monozygotic and dizygotic twins. We applied GCTA based on children's genotypes to detect genetic influence on family SES as well as genetic mediation of the effect of family SES on children's cognitive development.

## 2 Methods

### 2.1 Sample and genotyping

The sample was drawn from the Twins Early Development Study (TEDS), which is a multivariate longitudinal study that recruited over 11,000 twin pairs born in England and Wales in 1994, 1995 and 1996 (Haworth, Davis, & Plomin, 2013). TEDS has been shown to be representative of the UK population (Kovas, Haworth, Dale, & Plomin, 2007). The project received approval from the Institute of Psychiatry ethics committee (05/Q0706/228) and parental consent was obtained prior to data collection. Cognitive data and buccal DNA were available for 3747 11- and 12-year-old children (one twin per family), whose first language was English and had no major medical or psychiatric problems. From that sample, 3665 DNA samples were successfully hybridized to Affymetrix GeneChip 6.0 SNP genotyping arrays using standard experimental protocols as part of the WTCCC2 project (Trzaskowski et al., 2013b). In addition to nearly 700,000 genotyped SNPs, more than one million other SNPs were imputed using IMPUTE v.2 software (Howie, Donnelly, & Marchini, 2009). 3152 DNA samples (1446 males and 1706 females) survived quality control criteria for ancestry, heterozygosity, relatedness, and hybridization intensity outliers. To control for ancestral stratification, we performed principal component analyses on a subset of 100,000 quality-controlled SNPs after removing SNPs in linkage disequilibrium (*r*2 > 0.2). Using the Tracy–Widom Test, we identified 8 axes with *p* < 0.05, which were used as covariates in our GCTA analyses. This is standard procedure in genome-wide association analyses to avoid artificial associations due to ethnic or other types of population stratification (Trzaskowski et al., 2013b); correcting for these covariates is also standard in GCTA in order to avoid this source of genetic similarity among individuals in the population (Yang et al.).

The mean age of the sample at the first wave of assessment was 7.04 years (SE = 0.25) and 11.5 years (SD = 0.66) at the second wave. There were 2679 individuals with SES at 7 and 1897 with IQ at 7. The sample of individuals available for the covariance was 1750. In addition, there were 2319 individuals with IQ at age 12 for whom a total of 2013 had data for both SES at age 7 and IQ at age 12.

### 2.2 Measures

#### 2.2.1 Socioeconomic status (SES)

There is general consensus that a composite of variables including parental education and occupation represent SES better than any single indicator (White, 1982). To index family SES we used parental education and occupation assessed when children were age 2 and again when children were age 7. At age 2, SES was constructed from the first unrotated principal component, which explained more than 50% of the variance from a factor analysis conducted on five measures: father's highest educational qualification, father's occupation, mother's highest educational qualification, mother's occupation, and age of mother at birth of eldest child. The SES composite when children were age 7 was created similarly but without the variable of age of mother at birth of eldest child.

#### 2.2.2 General cognitive ability (IQ)

At ages 7 and 12, IQ was assessed from two verbal tests and two non-verbal tests. At age 7, the two verbal tests consisted of the Similarities subset and the Vocabulary subset from the WISC-III-UK. The two nonverbal tests were the Picture Completion subset from the WISC-III-UK and the Conceptual Grouping subset from the McCarthy Scales of Children's Abilities. At age 12, the verbal tests were Information (General Knowledge) and Vocabulary Multiple Choice subtests from WISC-III-PI. The two non-verbal reasoning tests were WISC-III-UK Picture Completion and Raven's Standard and Advanced Progressive Matrices. At age 7, testing was conducted by telephone (Petrill, Rempell, Oliver, & Plomin, 2002) and at age 12 testing was conducted over the Internet (Haworth et al., 2007).

#### 2.2.3 Composite measures for IQ

For each cognitive measure, outliers above or below 3 SD from the mean were excluded. Scores were regressed on sex and age and standardized residuals were derived and quantile normalized (Lehmann, 1975). Subsequently, composite measures for IQ were created as unit-weighted means requiring complete data for at least 3 of the 4 tests. All procedures were executed using R (www.r-project.org).

### 2.3 Statistical analyses

Genome-wide Complex Trait Analysis (GCTA). Conceptually GCTA compares a matrix of pairwise genomic similarity to a matrix of pairwise phenotypic similarity using a random-effects mixed linear model in a large sample of unrelated individuals (Yang et al., 2011). The matrix that holds genomic similarities between all individuals from the sample is known as the genetic relatedness matrix (GRM). Each value in the GRM is a mean of pairwise genetic similarities (weighted by allele frequency) from across all genetic markers genotyped on the SNP array. Even remotely related pairs of individuals are excluded so that chance genetic similarity is used as a random effect in a mixed linear maximum likelihood model to decompose phenotypic variance into genetic variance as captured by the additive effects of causal variants in linkage disequilibrium with SNPs genotyped on DNA arrays (Yang et al., 2010). For this reason, as a default, GCTA removes one individual from a pair whose genetic similarity is 0.025 or greater; a coefficient that approximates at least fifth degree relatives. The power of the method comes from comparing, not just two groups like MZ and DZ twins, but thousands of pairs of unrelated individuals. Nonetheless, GCTA requires samples of thousands of individuals because the method attempts to extract a small signal of genetic similarity from the massive noise of hundreds of thousands of SNPs. Software is available to calculate power for univariate and bivariate GCTA (http://spark.rstudio.com/ctgg/gctaPower/)(Visscher et al., 2013). For example, a sample of 3000 has 80% power to detect a GCTA heritability estimate of 30% and 50% power to detect a GCTA heritability estimate of 20%. For bivariate analysis, a sample of 3000 provides 80% power to detect a genetic correlation of 0.60 and 50% power to detect a genetic correlation of 0.45 when the GCTA heritability of one trait is 20% and the other is 30%.

In univariate analysis, the coefficients are estimated using residual maximum likelihood and the significance of genetic influence is inferred from the likelihood ratio test by comparing this model to a ‘null’ model of no genetic influence. Detailed description of the method can be found in GCTA publications (Yang et al., 2011). The bivariate method extends the univariate model by relating the pairwise genetic similarity matrix to a phenotypic covariance matrix between traits 1 and 2 (Lee, Yang, Goddard, Visscher, & Wray, 2012). The eight principal components described earlier were used as covariates in our univariate and bivariate GCTA analyses in order to attenuate the effects of ethnic and other forms of population stratification that could be read as genetic similarity, which is standard procedure in genome-wide analyses. As mentioned in the previous section, IQ scores were age- and sex-regressed prior to analysis.

## 3 Results

Using SNP genotypes from the children's DNA, we found significant genetic influence on their families' SES when the children were age 2 and age 7 (Table 1). The GCTA estimates of heritability were 18% at age 2 and 19% at age 7; the similarity of results at age 2 and 7 is not a foregone conclusion because the correlation between family SES at the two ages is 0.75. These are underestimates of true heritability because GCTA is limited to detecting genetic influence due to additive effects of the common SNPs that are on current DNA microarrays such as the Affymetrix 6.0 GeneChip used in our study. That is, nonadditive effects and rarer DNA variants not tagged by common SNPs are missed in GCTA analysis. A novel aspect of the present study is that children cannot cause family SES — their genotypes only reflect the causal genotypic factors responsible for their parents' education and occupation. For this reason, parental genotypes, not available in the present study, would be expected to yield a higher GCTA estimate of the parents' own education and occupation, which comprise SES. This was the case for a recent GCTA report of a component of SES, adult educational attainment (Rietveld et al., 2013).

**Table 1:** Univariate genome-wide complex trait analysis (GCTA) results (with standard errors) for family socioeconomic status (SES) when the children were age 2 and age 7.

	V(G)	V(e)	Vp	V(G)/Vp	Log L	Log L0	LRT	df	p	n
SES 2	.18(.12)	.80(.12)	.98(.03)	.19(.12)	− 1407.95	− 1409.23	2.56	1	0.05	2864
SES 7	.19(.12)	.79(.12)	.99(.03)	.20(.12)	− 1321.16	− 1322.45	2.57	1	0.05	2679

Annotation: V(G) — variance explained by genetic factors; V(e) — residual variance; Vp — phenotypic variance; V(G)/Vp — proportion of the phenotypic variance explained by genetic factors; Log L — log likelihood estimation of the model; Log L0 — log likelihood estimation of the null model (no genetic component); LRT — likelihood ratio test (approximated to a half of chi-square); df — degrees of freedom; values in parentheses are standard errors.

A strength of our child-based design using children's genotypes in GCTA analyses rather than that of their parents is that it captures the genetic influence of family SES on the children themselves. This feature of the design enables the second stage of our analyses in which we conducted bivariate GCTA to determine the extent to which the well known link between family SES and cognitive development – about 0.30 in meta-analyses (Sirin, 2005) and in the present study – is mediated genetically. Because bivariate GCTA focuses on the genetic covariance between family SES and children's IQ, our analysis is limited to TEDS children for whom data are available for both variables. Despite the smaller samples, the bivariate GCTA heritability estimates for family SES (Table 2) are similar to the univariate estimates (Table 1): 21% at age 7 and 23% at age 12. For children's IQ, we find heritabilities of 28% at age 7 and 32% at age 12. These results from our bivariate analysis between family SES and children's IQ replicate our previously reported results showing significant GCTA heritability in our TEDS sample at ages 7 and 12 (Plomin et al., 2013, Trzaskowski, Yang, Visscher and Plomin, 2013) as well as another study that reported significant GCTA heritability at age 11 (Davies et al., 2011).

**Table 2:** Bivariate GCTA results (with standard errors) between family SES when children were age 7 versus children's IQ at ages 7 and 12.

Variables	A						E				Vp_tr1	Vp_tr2	n_tr1/n_tr2
	V(G)_tr1	V(G)_tr2	C(G)_tr12	V(G)/Vp_tr1	V(G)/Vp_tr2	r_G_	V(e)_tr1	V(e)_tr2	C(e)_tr12	r_E_*			
SES 7 – IQ 7	.21(.12)	.28(.17)	.29(.11)	.21(.12)	.28(.17)	1.00(.47)	.78(.12)	.72(.17)	.03(.11)	.04(.14)	.99(.03)	1(.03)	2679 1897
SES 7 – IQ 12	.23(.12)	.32(.14)	.18(.10)	.24(.12)	.32(.14)	0.66(.31)	.76(.12)	.68(.14)	.14(.10)	.20(.12)	.99(.03)	.99(.03)	2679 2319

Annotation: V(G) — variance explained by genetic factors for trait 1 and trait 2 (tr1, tr2); C(G) — covariance between trait 1 and 2 explained by genetic factors; V(e) — residual variance for trait 1 and trait 2; C(e) — residual covariance between trait 1 and trait 2; Vp — phenotypic variance for trait 1 and trait 2; V(G)/Vp — proportion of the phenotypic variance explained by genetic factors for trait 1 and trait 2; r_G_ — genetic correlation between trait 1 and trait 2 (constrained between 0 and 1); n — number of individuals with data for both trait 1 and trait 2; values in parentheses are standard errors.

*The current version of GCTA does not report the residual correlation or its standard error. The residual correlation was derived here from the GCTA estimates using the following algorithm: C(e)_tr12/(√V(e)_tr1 * √V(e)_tr2), whereas the standard error was calculated using: Var(re) = re * re * (VarVe1/(4*Ve1*Ve1) + VarVe2/(4*Ve2*Ve2) + VarCe/(Ce*Ce) + CovVe1Ve2/(2*Ve1*Ve2) - CovVe1Ce/(Ve1*Ce) - CovVe2Ce/(Ve2*Ce)); SE(re) = sqrt[Var(re)], where re is the residual correlation, Ve1 is the residual variance for trait 1, Ce is the residual covariance between two traits, VarVe1 is the sampling variance for Ve1 (residual variance for trait 1), VarCe is the sampling variance for Ce, CovVe1Ve2 is the sampling covariance between Ve1 and Ve2, and CovVe1Ce is the sampling covariance between Ve1 and Ce.

The key result for the bivariate GCTA analysis is the genetic correlation, which indicates the extent to which the same genes affect family SES and children's IQ. The genetic correlation between family SES at age 7 and children's IQ at age 7 is near unity, indicating that the same genes affect both variables (Table 2). Despite the large standard error for GCTA estimates of genetic correlations, the genetic correlation is significantly greater than zero and not significantly lower than 1.0. We also conducted bivariate GCTA for family SES at age 7 and children's IQ at age 12 (family SES was not assessed at age 12). The GCTA genetic correlation was 0.66, which was again significantly greater than zero and not significantly lower than 1.0. Thus, these GCTA genetic correlations indicate that the same genes are largely responsible for genetic effects on family SES and children's IQ. This finding implies that when genes associated with children's IQ are identified, the same genes will also be likely to be associated with family SES. Although GCTA estimates of genetic variance and genetic covariance are biased in that they underestimate heritability to the extent that nonadditive effects and rare alleles are not included in the estimate, GCTA estimates of genetic correlations are unbiased because they are derived from the ratio of genetic covariance to genetic variance so that the bias in the numerator and denominator cancel out (Trzaskowski et al., 2013a).

Bivariate GCTA analysis also indicates the extent to which the phenotypic covariance between family SES and children's IQ is mediated genetically. The phenotypic correlation between family SES at age 7 and children's IQ at age 7 is 0.31. The genetic contribution to this covariance is 0.29 (Table 2). In other words, 94% of the correlation between family SES and children's IQ is mediated genetically. For family SES at age 7 and children's IQ at age 12, 56% of the phenotypic correlation of 0.32 is mediated genetically. The large standard errors for the estimates of genetic correlations suggest that replication is needed before interpreting the difference between the 94% versus 56% results for IQ at ages 7 and 12, respectively. However, if the difference is real, one possible explanation is that, although the phenotypic correlations between age-7 SES and IQ at ages 7 and 12 do not change, the lower genetic contribution to the SES-IQ correlation at age 12 might reflect increased environmental influence outside the family (e.g., peers, teachers).

**Fig. 1:**
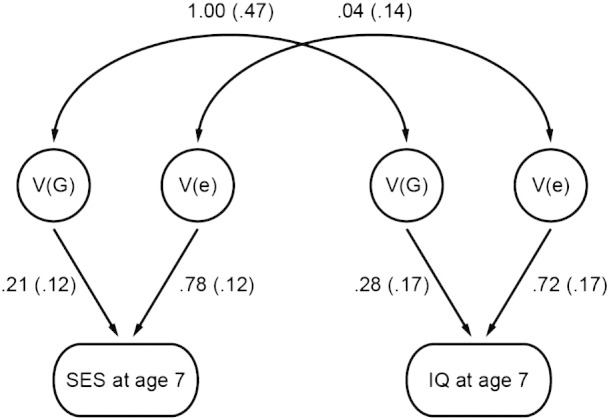
Genetic influence is significant and substantial on family SES and children's IQ and completely accounts for the association between family SES and children's IQ. Although this model looks like a path model depicting the results of a twin study, the within-family twin design cannot be used to assess between-family environmental measures such as family-level SES as in the present study. This model describes GCTA results based on DNA of unrelated children. The top row of numbers indicates genetic and environmental correlations, respectively. The bottom row of numbers indicates the proportion of variance in SES and in IQ that can be attributed to genetic and non-genetic factors. (That is, these are not standardized partial regressions that need to be squared to estimate variance explained.)

In summary, genetic influence is significant and substantial on family SES, on children's IQ, and on the association between family SES and children's IQ. Fig. 1 summarizes the results for family SES at age 7 and children's IQ at age 7, incorporating data from Table 2.

## 4 Discussion

Our analysis provides the first DNA-based evidence that the well documented association between family SES and children's cognitive development, routinely interpreted as an environmental effect, is substantially mediated by genetic factors. Previous quantitative genetic research, largely using the twin design, has shown that most ‘environmental’ measures involve significant genetic influence and that associations between these environmental measures and children's development are mediated genetically (Plomin, 1994, Plomin et al., 2013, Vinkhuyzen et al., 2010). GCTA adds importantly to this body of research in two ways. First, because it uses DNA alone, GCTA sidesteps concerns about the twin design such as the equal environments assumption (Plomin, DeFries, Knopik, & Neiderhiser, 2013), which might be especially relevant to measures of family environment (Power et al., 2013). Second, many important environmental factors such as family SES cannot be studied using the twin design because they operate between families (family general) rather than within families (child specific). GCTA can be used to study between-family variables.

The main limitation of this study is the sample size. Although a sample of 3000 unrelated children with genome-wide genotypes and data on IQ and family SES is large by many standards, as noted earlier, GCTA has daunting demands for power. Our sample size is just on the cusp of being able to detect as significant the GCTA heritabilities of family SES, which is about 20%. It should be reiterated that GCTA heritability estimates are lower-limit estimates of twin heritability because GCTA is limited to detecting the additive effects of the common SNPs used in our genome-wide genotyping. On the other hand, because we found such high genetic correlations, we have good power to detect them. As noted earlier a sample of 3000 provides 80% power to detect a genetic correlation of 0.60.

Although these results are surprising and provocative, they do not in any way support the misguided notion that heritability implies immutability. Nor do any specific policies necessarily follow from finding genetic influence on family SES and its correlation with children's cognitive development because policies depend on values. However, our results do underline the need to consider nature as well as nurture when drawing policy implications from correlations between ostensible environmental measures and children's developmental outcomes. Specifically, our results bear on the extensive debate about social mobility, which has largely ignored the fact that parents and their offspring are genetically related (Saunders, 2012). Indeed, the correlation between parent and offspring SES is used as an index of intergenerational social mobility because it is assumed that SES advantages are transmitted environmentally from parent to offspring (Breen & Jonsson, 2005). For this reason, lower parent–offspring correlations are thought to indicate social mobility. From this environmental perspective, it follows that equal educational and occupational opportunities will result in equal outcomes between children so that parental SES would no longer have any effect on children's cognitive development, education or occupation. On the contrary, taking genetics into account suggests that *higher* parent–offspring correlations indicate social mobility. To the extent that genetics is important, parents and their offspring will be correlated; removing environmental sources of inequality will not remove this fundamental resemblance between parents and offspring.

More broadly, it should be recognized that from a genetic perspective, equal opportunity will result in relatively greater genetic influence, as reflected in greater parent–offspring correlations: As environmental differences diminish, variation that remains between children in their outcomes will be due to a greater extent to their genetic differences. In other words, heritability can be viewed as an index of meritocratic social mobility.

## Author contributions

M.T., P.D and R.P. designed the study and analyses. M.T. and A.M. analyzed the data. All authors contributed to discussions of the results and their implications and to writing and revising the paper. R.P. directs TEDS.

## Competing financial interests

The authors declare no competing financial interests.

## References

[bb0030] Bradley R.H., Corwyn R.F. (2002). Socioeconomic status and child development. Annual Review of Psychology.

[bb0095] Breen R., Jonsson J.O. (2005). Inequality of opportunity in comparative perspective: Recent research on educational attainment and social mobility. Annual Review of Sociology.

[bb0050] Davies G., Tenesa A., Payton A., Yang J., Harris S.E., Liewald D. (2011). Genome-wide association studies establish that human intelligence is highly heritable and polygenic. Molecular Psychiatry.

[bb0100] Haworth C.M.A., Davis O.S.P., Plomin R. (2013). Twins Early Development Study (TEDS): A genetically sensitive investigation of cognitive and behavioral development from childhood to young adulthood. Twin Research and Human Genetics.

[bb0135] Haworth C.M.A., Harlaar N., Kovas Y., Davis O.S.P., Oliver B., Hayiou-Thomas M.E. (2007). Internet cognitive testing of large samples needed in genetic research. Twin Research and Human Genetics.

[bb0115] Howie B.N., Donnelly P., Marchini J. (2009). A flexible and accurate genotype imputation method for the next generation of genome-wide association studies. PLoS Genetics.

[bb0005] Kendler K.S., Baker J.H. (2007). Genetic influences on measures of the environment: A systematic review. Psychological Medicine.

[bb0105] Kovas Y., Haworth C.M.A., Dale P.S., Plomin R. (2007). The genetic and environmental origins of learning abilities and disabilities in the early school years. Monographs of the Society for Research in Child Development.

[bb0065] Lee S.H., Yang J., Goddard M.E., Visscher P.M., Wray N.R. (2012). Estimation of pleiotropy between complex diseases using single-nucleotide polymorphism-derived genomic relationships and restricted maximum likelihood. Bioinformatics.

[bb0140] Lehmann E.L. (1975). Nonparametrics: Statistical methods based on ranks.

[bb0130] Petrill S.A., Rempell J., Oliver B., Plomin R. (2002). Testing cognitive abilities by telephone in a sample of 6-to 8-year olds. Intelligence.

[bb0015] Plomin R. (1994). Genetics and experience: The interplay between nature and nurture.

[bb0010] Plomin R., Bergeman C.S. (1991). The nature of nurture: Genetic influence on "environmental" measures. The Behavioral and Brain Sciences.

[bb0025] Plomin R., DeFries J.C., Knopik V.S., Neiderhiser J.M. (2013). Behavioral genetics.

[bb0055] Plomin R., Haworth C.M.A., Meaburn E.L., Price T.S., Davis O.S.P., Wellcome Trust Case Control Consortium 2 (2013). Common DNA markers can account for more than half of the genetic influence on cognitive abilities. Psychological Science.

[bb0085] Power R.A., Wingenbach T., Cohen-Woods S., Uher R., Ng M.Y., Butler A.W. (2013). Estimating the heritability of reporting stressful life events captured by common genetic variants. Psychological Medicine.

[bb0060] Rietveld C.A., Medland S.E., Derringer J., Yang J., Esko T., Martin N.W. (2013). GWAS of 126,559 individuals identifies genetic variants associated with educational attainment. Science.

[bb0090] Saunders P. (2012). Social mobility delusions.

[bb0070] Sirin S.R. (2005). Socioeconomic status and academic achievement: A meta-analytic review of research. Review of Educational Research.

[bb0075] Trzaskowski M., Yang J., Visscher P.M., Plomin R. (2013). DNA evidence for strong genetic stability and increasing heritability of intelligence from age 7 to 12. Molecular Psychiatry.

[bb0080] Trzaskowski M., Davis O.S.P., DeFries J.C., Yang J., Visscher P.M., Plomin R. (2013). DNA evidence for strong genome-wide pleiotropy of cognitive and learning abilities. Behavior Genetics.

[bb0110] Trzaskowski M., Eley T.C., Davis O.S.P., Docherty S.J., Hanscombe K.B., Meaburn E.L. (2013). First genome-wide association study on anxiety-related behaviours in childhood. PLoS ONE.

[bb0020] Vinkhuyzen A.A.E., van der Sluis S., de Geus E.J.C., Boomsma D.I., Posthuma D. (2010). Genetic influences on 'environmental' factors. Genes, Brain, and Behavior.

[bb0145] Visscher P.M., Hemani G., Vinkhuyzen A.A.E., Chen G.-B., Lee S.H., Wray N.R. (2013). Statistical power to detect genetic (co)variance of complex traits using SNP data in unrelated samples. PLoS Genetics.

[bb0125] White K.R. (1982). The relation between socioeconomic status and academic achievement. Psychological Bulletin.

[bb0035] Yang J.A., Lee S.H., Goddard M.E., Visscher P.M. (2011). GCTA: A tool for genome-wide complex trait analysis. American Journal of Human Genetics.

[bb0040] Yang J., Benyamin B., McEvoy B.P., Gordon S., Henders A.K., Nyholt D.R. (2010). Common SNPs explain a large proportion of the heritability for human height. Nature Genetics.

[bb0120] Yang J., Weedon M.N., Purcell S. (2011). Genomic inflation factors under polygenic inheritance. European Journal of Human Genetics.

